# Exploring Multiple Goals Balancing in Complex Problem Solving Based on Log Data

**DOI:** 10.3389/fpsyg.2019.01975

**Published:** 2019-09-27

**Authors:** Yan Ren, Fang Luo, Ping Ren, Dingyuan Bai, Xin Li, Hongyun Liu

**Affiliations:** ^1^School of Psychology, Beijing Normal University, Beijing, China; ^2^Collaborative Innovation Center of Assessment Towards Basic Education Quality, Beijing Normal University, Beijing, China

**Keywords:** complex problem solving, multiple goals balancing, log data analysis, educational data mining, K-means cluster analysis

## Abstract

Multiple goals balancing is an important but not yet fully validated dimension of complex problem solving (CPS). The present study used process data to explore how solvers clarify goals, set priorities, and balance conflicting goals. We extracted behavioral indicators of goal pursuit from the log data of 3,201 students on the third subtask of the “Ticket” task in the PISA 2012 CPS test. Cluster analysis was used to identify 10 groups that varied in goal pursuit behavior. Logistics and least-squares regression analysis were used to explore how goal pursuit affected task scores and CPS proficiency. The results showed that competent solvers clarified goals and weighed priorities more effectively. They also made trade-offs between conflicting goals. The importance of theoretically-driven log data analysis and coping strategies in the face of multiple goals conflict scenarios was discussed.

## Introduction

Science and technology are developing in the current information explosion era. People are facing an increasing number of complex problems in daily life, many of which involving the simultaneous pursuit of multiple goals. Therefore, complex problem solving (CPS) becomes common in real life, such as the use of complex technology (e.g., mobile phones, personal computers, and vending machines), the management of complex organizations (e.g., companies and departments), and the prediction of complex environments (e.g., weather and stock prices; Funke, 2003, 2010).

Complex problem solving refers to successful interaction with a dynamic task environment, wherein all or some rules in the environment can only be learned by exploring and integrating information (Buchner, 1995). Many researchers have suggested that CPS should be assessed in a simulated problem scenario (a complex system) where has a plurality of variables. In the scenario, solvers are asked to manipulate some of the variables to explore effective rules of describing relationships among all variables (knowledge acquisition), and then solvers need to use the learned knowledge of rules to achieve specific goals (knowledge application; Funke, 2001).

### Multiple Goals in Complex Problem Solving

Blech and Funke (2010) verified that the presence of conflicting goals affects the difficulty of a complex system. They found that increasing the number of goals in CPS – especially with respect to conflicting goals – increases the cognitive and emotional challenges faced by solvers (Funke, 1992). Therefore, in the case of complex problems involving multiple goals, solvers may not be able to fully account for each goal. Thus, they might weigh the priority of each. They may first achieve one goal and then find the next one; they may sacrifice one goal in exchange for another; or they may choose to achieve a complementary goal. These strategies emphasize the importance of goal priority (Funke, 2010). Dörner and Kreuzig (1983) proposed that operative intelligence involves the skills of goal elaborating and goal balancing. Later, Dörner proposed the CPS action theory, which divides the CPS solution process into six characteristic phases, of which one is exploring and determining important parts of the system (e.g., such as defining and balancing conflicting goals; Dörner and Wearing, 1995).

The five-dimensional model of CPS consists of system exploration, information reduction, model formation, control considering dynamic change, and prioritization of goals, in which the ability to clarify, prioritize, and balance goals is an important dimension (Funke, 2001; Fischer et al., 2012; Greiff and Fischer, 2013; Schoppek and Fischer, 2015; Herde et al., 2016). However, when assessing CPS, researchers usually develop complex systems based on simplified models. For example, complex systems like Genetic Lab (Sonnleitner et al., 2012), MicroDYN and MicroFIN (Greiff et al., 2012) were developed based on the three-dimensional model comprising information retrieval, model building, and forecasting or the two-dimensional model including knowledge acquisition and knowledge application. The assessment of problem solving in PISA 2012^1^ is based on a four-dimensional CPS model consisting of exploring and understanding, representing and formulating, planning and executing, and monitoring and reflecting (OECD, 2013). The use of this simplified model neglects other CPS dimensions that have been raised in the literature. For example, no researchers have developed complex systems that directly measure the skill of multiple goals balancing, nor have any researchers attempted to extract behavioral indicators from the log files of a complex system to evaluate solvers’ competency in this skill.

### Data Mining of the Log Data

Analysis of log data has become an important method for revealing how CPS proficiency might be improved (Herde et al., 2016; Dörner and Funke, 2017). Comparing to results data, process data contain more information about the problem solving process, which can better represent solvers’ actual CPS proficiency. Some researchers have attempted to conduct preliminary analyses of log data recorded by complex systems. It is found that the “vary one thing at a time” (VOTAT) strategy is an effective problem solving strategy in complex systems (Vollmeyer et al., 1996; Wüstenberg et al., 2014). Greiff et al. (2016) collected the log data of MicroDYN and counted solvers’ non-interfering observations, intervention frequency, VOTAT strategy, and time on task. In this study, solvers were instructed to manipulate the input variables and then to click on “apply” to activate it, and each round represented an intervention. The results showed that good problem solvers were not only good at using VOTAT, but also had moderately frequent intervention, highly frequent non-intervention observation, and a moderate response time; poor problem solvers tended to demonstrate little intervention or to constantly intervene.

However, most of the process indicators extracted from log data proposed by the previous research are simple indicators (e.g., time on task, intervention frequency, etc.) and fail to delve into meaningful CPS behavior sequences. To date, VOTAT is the only CPS strategy based on the behavior sequences that has been verified by log data analysis (Kröner et al., 2005; Wüstenberg et al., 2012; Müller et al., 2013; Greiff et al., 2015).

The purpose of the present study is to test and supplement the theoretical discussion of multiple goals balancing in the literature based on the meaningful CPS behavior sequences contained in the log data.

### This Study

Although researchers generally believe that multiple conflicting goals is an important feature of CPS systems, not all CPS systems have this feature, such as MicroDYN (Schoppek and Fischer, 2015). If the problem solving goals of a complex system are clear and equally important or independent and non-conflicting, solvers’ ability to balance multiple goals cannot be assessed. After considering the above-mentioned issues, the present study selected a proper CPS task that can meet the research purpose. Log data were analyzed to explore solvers’ processes of clarifying goals, setting priorities, and making trade-offs between conflicting goals. Thereby, the study aimed at confirming the important role of multiple goals balancing in CPS and increasing emphasis placed on this skill.

With the overarching goal of exploring the role of multiple goal balancing ability in CPS, the research questions were as follows:

(1) When solving complex problems, how do solvers clarify and weigh goal priorities to achieve better scores?
(2) Which goal pursuing strategies are more productive to solving a complex problem?

## Materials and Methods

### Task Descriptions

**FIGURE 1:**
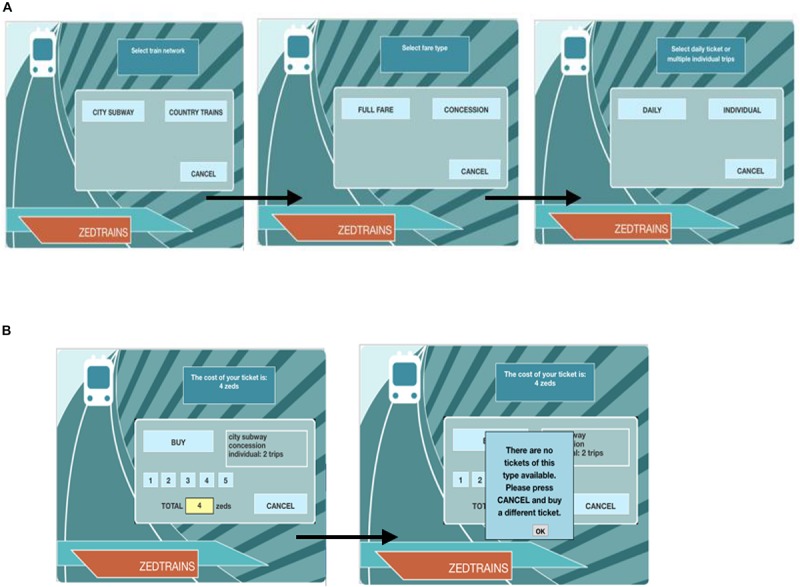
Interface of the “Ticket” task. **(A)** Selecting stage. **(B)** Purchasing stage.

In the study, data from the “Ticket” task of the PISA 2012 CPS log data were selected as the analytical subject. This task requires solvers to purchase a ticket using a virtual ticket vending machine. Ticket type is determined by three attributes [as shown in Figure 1A: train network (city subway or country train), fare type (full fare or concession), and trip (daily or individual)]. Daily tickets can be used an unlimited number of times on the day of purchase. Individual tickets can be used on different dates. If the latter is selected, the number of trips must be determined (from 1 to 5). Therefore, solvers have a total of 2 × 2 × (1 + 5) = 24 ticket types to choose from. When the ticket type is determined, the ticket price in zed (virtual currency unit) will be shown on the vending machine (see Figure 1B). Solvers then have two options: purchase the ticket or cancel the purchase and return to the initial selection screen.

The subject of the present study was the third subtask of the “Ticket” task, wherein solvers must purchase a city subway ticket that includes two trips. More than one choice is available to meet the ride need. Thus, solvers must consider the two goals of ride demand and price discount simultaneously, in order to find an optimal ticket. However, when solvers choose to purchase concession tickets, they receive notice that “There are no tickets of this type available. Please press CANCEL and buy a different ticket.” Because solvers only get feedback that the ticket is not available and receive no further information about the reasons why, they must try more ticket types until they are successful in securing a ticket. Thus, the third goal of the subtask is to find an available ticket.

The optimal purchase plan meets the three goals simultaneously: it satisfies the ride demand (demand goal), has the lowest price (price goal), and is available (availability goal). Since no concession tickets meet the needs of city travel, if solvers always work toward the price goal; they will repeatedly encounter a situation in which they cannot buy a concession ticket. Therefore, solvers must buy a slightly more expensive ticket in order to perform the task. Therefore, there is a direct conflict between the price goal and the availability goal. The demand and availability goals are superior to the price goal, and solvers should give priority to achieving the first two goals before striving to fulfill the price goal.

In summary, this third subtask of the “Ticket” task contains multiple conflicting goals with varying priority. Therefore, the log data of this subtask were deemed suitable for exploring solvers’ ability to balance multiple goals in CPS.

### Log Data Sample

The study was based on a secondary analysis of previously collected and publicly available data. The data selected from the PISA 2012 CPS log data were de-identified. The fare system of subway ticket vending machines is common in developed countries. We selected students from six developed countries: Austria, Japan, Australia, Ireland, Germany, and France. Because it is reasonable to believe that they share similar behavioral patterns given that their countries have similar economic backgrounds, and the students could be coded with the same coding scheme. A total of 40,217 students in these countries participated in the test, and the log data of 3,896 (9.69%) students were made available for analysis.

**FIGURE 2:**
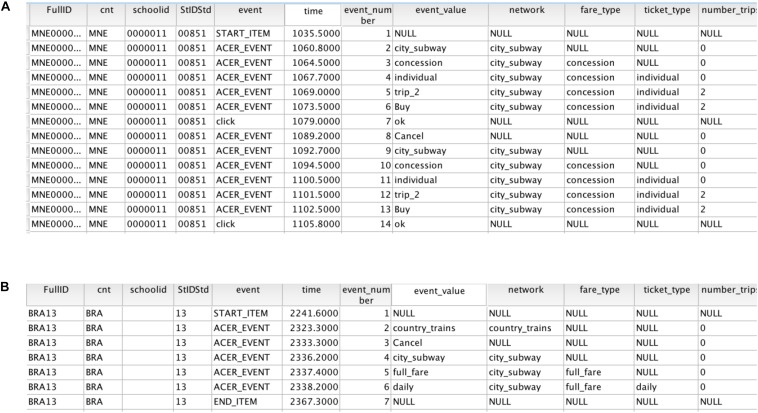
Example of log data for the third subtask of the “Ticket” task. **(A)** Example of complete plan log data. **(B)** Example of incomplete plan log data. The variables (from left to right) are full ID code, country code, school code, student code, event type (task start, task end, or intermediate event), time point of the event, serial number of the event, value of the event input variable, type of train network, type of price, type of ticket, and number of trips.

Partial log data are displayed in Figure 2A, below. Each row records an operation of an individual solver. Solvers’ cancelation or purchase operations after generating a complete ticket purchase were treated as segmentation marks between plans, and operations were divided into different ticket purchase plans. For example, Figure 2 shows the first 14 operations of a particular student. In the first six trials, the student attempted to purchase the ticket plan of (city subway, concession, individual ticket, two trips), and then canceled the purchase. This represented a complete purchase plan. The last six actions constitute the solver’s second purchase plan. In addition, a purchase plan may be incomplete. For example, the first three operations in Figure 2B showed that he/she chose the country train, and then clicked the cancel button. Because the solver clicked CANCEL without selecting fare type and trip, this purchase plan was considered as incomplete. A total of 5,933 incomplete operations were excluded from the log data, resulting in 113,707 actions in the final data set (95.04%).

Six hundred and ninety-five students with less than two plans after the first purchase plan were excluded because there were not enough plans to analyze their competence of balancing goals. Data from the remaining 3,201 students (82.16%) were used in the final analysis. Among these students, there were 1,594 males and 1,607 females, with an average age of 15.82 years (*SD* = 0.39).

### Indicators of Goal Pursuit

In order to evaluate whether problem solvers clearly identified and pursued the goals, two psychometricians and three undergraduate psychology students were invited to identify solvers’ behaviors that can represent goal pursuit. If no sufficient reasons can support the behavior is goal pursuit, then the conclusion cannot be made. This means that goal pursuit was defined as a deliberate strategy that solvers pursue rather than the solvers’ willingness in pursing the goal.

The following coding schemes were ultimately developed:

(1) Demand goal pursuit: If the plan met the ride demand as required by the task (city subway with two trips or an unlimited ticket), it would be coded as “1” (pursuing the demand goal); otherwise, it would be coded as “0” (abandoning the demand goal).
(2) Price goal pursuit: Solvers could only see the ticket price (the only output variable of the “Ticket” task) after completing their ticket selection. Thus, if a solver canceled the ticket after seeing the price, we would conclude that the solver was not satisfied with the price. That is, if the plan ended in “cancel,” the plan would be coded as “1” (pursuing the price goal).

If the plan ended with “purchase,” further judgment would be needed to determine whether the price of the plan was the cheapest among the available plans known to the solver (all previous plans except failed ones). If all of the plans that were known to the solver were failed, because there were no enough evidences to illustrate if the solver considered the price, we applied strict rules, which was the plan had to be the cheapest among all previous failed plans. In either scenario, if the price of the plan ended with “purchase” was the cheapest, then it would be coded as “1” (pursuing the price goal), otherwise it would be coded as “0” (abandoning the price goal).

(3) Availability goal pursuit: Participants would know that some tickets could not be bought but had to be got through strategic plans only after they clicked “purchase” at the first time but did not receive tickets, so all the plans before the first purchase plan would be coded as missing values for the unawareness of the availability goal.

When the solver found that the ticket is not exist, he/she usually changed one attribute or just changed the number of trips. However, this change could not reveal the solver’s deliberate efforts to avoid failure. The availability goal pursuit indicator should reflect the search strategy of available ticket type between attributes rather than within attributes. Therefore, we used three consecutive plans to code the indicator. Plan adjustment made on the basis of a solver’s initial purchase failure would be recorded as “Adjustment 1–2,” and the next plan adjustment would be recorded as “Adjustment 2–3.” When “Adjustment 1–2” and “Adjustment 2–3” pertained to different ticket attributes, the third plan would be coded as “1,” (pursuing the availability goal); otherwise, it would be coded as “0” (abandoning the availability goal). Similarly, “Adjustment 3–4” and so on would be coded according to the previous “Adjustment.”

The ratios of the number of the plans pursued for each of the three goals to the total number of plans would be considered goal pursuit indicators. We developed an autoscoring program in the R language that divided the operations into mark plans, judged whether the plans were complete, and coded the goal pursuit of plans. The autoscoring program in R has been double-checked by two undergraduate psychology students.

### Statistical Analysis

Cluster analysis was carried out on the goal pursuit indicators in order to identify groups with different set goal priorities. These groups were then compared to the task score used in the present study (with “1” meaning success and “0” meaning failure in problem solving) and CPS proficiency (solvers’ ability estimated from their performance on the PISA 2012 CPS test) in order to explore whether groups with better priority setting showed better performance. We chose the partitioning around medoids (PAMs) algorithm for cluster analysis, because it was more robust than K-Means against noise and outliers (Kaufman and Rousseeuw, 1987, 2009). The package ‘FPC’ (‘Flexible Procedures for Clustering’) of the statistical software R 3.4.4. was used to carry out PAM algorithm (Hennig, 2007).

Regressions analyses were used to analyze the effects of goal pursuit indicators and their interactions on problem solving performance. A simple slope test of the interaction was applied to explore the impact of different combinations of the three goal pursuit behaviors on problem solving and to probe problem solvers’ strategies for choosing among conflicting goals. The standardized z-scores of goal pursuit indicators and their interactions were predictors, and the task score and CPS proficiency on the PISA 2012 test were outcome variables. Regression analyses were performed using SPSS 23.0 (IBM, 2015).

## Results

### Descriptive Statistics

The descriptive statistics are presented in Table 1. Results showed that the third “Ticket” subtask score was moderately correlated with CPS proficiency (*r* = 0.39). Three goal pursuit indicators were correlated with task score and CPS proficiency.

**TABLE 1:** Descriptive statistics of variables.

**Variable**	***M***	***SD***	**Minimum**	**Maximum**	**1**	**2**	**3**	**4**
(1) Task score	0.63	0.48	0	1	–			
(2) CPS proficiency	544.06	86.75	214.9	802.5	0.39^∗∗^	–		
(3) Demand goal pursuit (%)	90.12	13.65	0	100	0.38^∗∗^	0.23^∗∗^	–	
(4) Price goal pursuit (%)	68.30	23.18	0	100	−0.04^∗^	–0.02	0.10^∗∗^	–
(5) Availability goal pursuit (%)	61.91	27.79	0	100	0.25^∗∗^	0.09^∗∗^	–0.05^∗∗^	–0.31^∗∗^

*^∗^*p* < 0.05, ^∗∗^*p* < 0.01. The columns names 1,2,3,4 refer to the four variables in the rows.*

### Cluster Analysis of Goal Pursuit Indicators

The PAM algorithm was applied to cluster the dataset. The numbers of clusters were determined at 10 when the average silhouette width reached the maximum value. Table 2 lists the number of people in each group, within-cluster sum of squares and their average values for task score, CPS proficiency, and the three goal pursuit behaviors (columns 6–8, respectively) and their z-scores (columns 9–11, respectively).

**TABLE 2:** Cluster results of goal pursuit.

**Group**	**Number of people**	**Within-cluster sum of squares**	**Task score**	**CPS proficiency**	**Demand goal (%)**	**Availability goal (%)**	**Price goal (%)**	**Demand goal (z)**	**Availability goal (z)**	**Price goal (z)**
1	447	197.61	0.88	557.04	99.37	98.99	51.03	0.68	1.33	–0.74
2	411	214.50	0.88	575.15	98.19	79.16	93.13	0.59	0.62	1.07
3	73	181.26	0.82	561.16	99.57	92.45	4.88	0.69	1.10	–2.73
4	501	153.07	0.81	563.47	97.46	56.07	61.99	0.54	–0.21	–0.27
5	558	375.86	0.61	542.74	95.98	35.9	83.63	0.43	–0.94	0.66
6	369	313.75	0.50	530.97	82.29	45.59	45.27	–0.57	–0.59	–0.99
7	456	204.50	0.43	528.00	76.9	64.05	75.73	–0.97	0.08	0.32
8	117	84.88	0.24	494.11	67.32	91.23	34.78	–1.67	1.05	–1.44
9	117	307.71	0.24	498.81	48.48	76.34	78.26	–3.05	0.52	0.43
10	152	104.38	0.18	507.83	99.15	5.87	97.72	0.66	–2.02	1.27
overall	3201	2137.52	0.63	544.06	90.12	61.91	68.30	0	0	0

The z-scores of the three goal pursuit behaviors were compared across groups. The results showed that the three groups with the highest task scores showed positive demand and availability goals pursuit (0.68, 0.59, and 0.69; and 1.33, 0.62, and 1.10, respectively). In other groups, at least one of the demand and availability goals was negative. This showed that successful problem solvers worked hard on both of these goals. The price goal pursuit of Groups 1, 3, and 4 were negative (−0.74, −2.73, and −0.27, respectively), and that of Group 2 was positive (1.07), which indicated that price goal pursuit was not important for solving the problem.

The task score of Group 5 (0.61) was 0.2 lower than that of Group 4 (0.81). In this group, demand goal pursuit was positive, but availability goal pursuit was negative (−0.94), that is the availability goal was ignored, and the price goal was much more highly prioritized than that in Group 4. Group 10 pursued a higher demand goal but neglected the availability goal (−2.02); it also excessively pursued the price goal (1.27), which greatly reduced the task score (0.18). This showed that pursuit of the demand goal on its own could not effectively solve the problem; rather, solvers had to also pursue the availability goal, without strongly pursuing the price goal.

The demand goal pursuit of all other groups was negative, and this affected their task scores (0.24–0.50) (e.g., Groups 6–9).

### Logistic Regression of Goals Pursuit on Task Score

Logistic regression was used to test whether task score could be predicted by three types of goal pursuits and their interactions. The results of the Omnibus Test compared to the previous model were significant (χ^2^ = 770.433, *df* = 3, *p* < 0.001; χ^2^ = 112.543, *df* = 2, *p* < 0.001) when adding all goal pursuits and their two-way interaction terms into the model in sequence. The results showed that the availability goal pursuit and the demand goal pursuit as well as the interactions between the availability goal pursuit and the other two goal pursuits, respectively, were significant and had effects that differ from zero.

The Nagelkerke *R*^2^ of the final model was 0.329, demonstrating a medium effect on task score by goal pursuit. The prediction accuracies of the correct and incorrect responses were 87.2 and 54.7%, respectively, and total accuracy was 75.3% (see Table 3).

**TABLE 3:** Logistic regression of goal pursuit on task score.

		**Correct or**		
		**incorrect**		
		**prediction**		**Predicted**
		**results**		**accuracy (%)**
		**0**	**1**	
Observation data of correct and incorrect answers	0	644	533	54.7
	1	259	1765	87.2
	Overall			75.3

Demand goal pursuit (*B* = 1.041, Wald χ^2^_(*df* = 1)_ = 394.670, *p* < 0.001) and availability goal pursuit (*B* = 0.702, Wald χ^2^_(*df* = 1)_ = 198.667, *p* < 0.001) significantly positively predicted task score. The demand goal resulted in a larger regression coefficient than the availability goal (see Table 4).

**TABLE 4:** Logistic regression coefficient of goal pursuit on task score.

**Predictor**	**Model 1**			**Model 2**		
	***B***	***SE***	**Wald χ^2^**	***B***	***SE***	**Wald χ^2^**
Demand	1.013	0.049	425.54^∗∗∗^	1.041	0.052	394.670^∗∗∗^
Availability	0.726	0.047	235.425^∗∗∗^	0.702	0.050	198.667^∗∗∗^
Availability * demand				0.277	0.055	25.187^∗∗∗^
Availability * price				0.319	0.042	56.857^∗∗∗^
Nagelkerke *R*^2^	0.292			0.329		
The Omnibus Test	770.433(3)			112.543(2)		
(χ^2^/df)						

*^∗∗∗^*p* < 0.001. The variables not significant was excluded in the model.*

**FIGURE 3:**
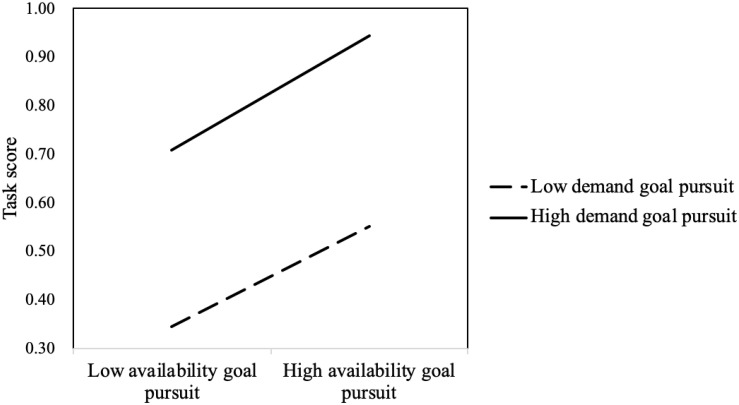
Interaction between availability goal pursuit and demand goal pursuit.

The interaction between demand goal pursuit and availability goal pursuit was significant (*B* = 0.277, Wald χ^2^_(*df* = 1)_ = 25.187, *p* < 0.001). The results of the simple slope test of availability goal pursuit at high/low demand goal pursuit (the mean plus/minus a standard deviation) are shown in Figure 3. On the whole, solvers with high demand goal pursuit scored higher than those with low demand goal pursuit. This suggested that the demand goal was more important than the availability goal. For those with high demand goal pursuit, availability goal pursuit significantly positively predicted task score to a greater extent (*B* = 0.559, *p* < 0.001). For those with low demand goal pursuit, availability goal pursuit significantly positively predicted task score to a smaller extent (*B* = 0.246, *p* < 0.001) (see Figure 3). If the solver pursuing the demand goal pursued the availability goal at the same time, he/she would efficiently improve task scores and solve the problem best. However, if the solver neglected the demand goal, even though pursuing availability goal was beneficial to improve task score, the improvement would be limited.

**FIGURE 4:**
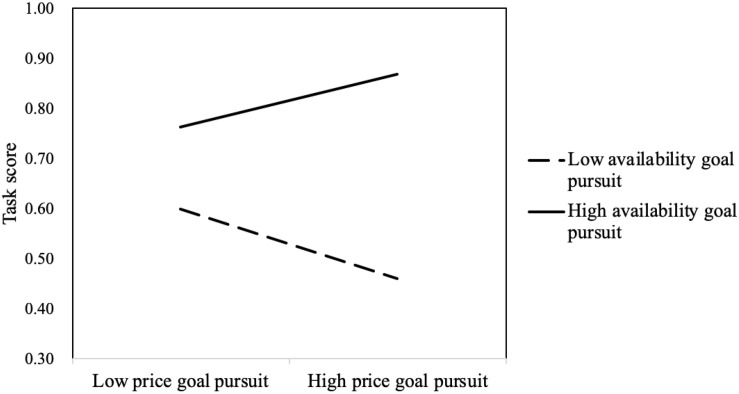
Interaction between availability goal pursuit and price goal pursuit.

The interaction between price goal pursuit and availability goal pursuit was significant (*B* = 0.319, Wald χ^2^_(*df* = 1)_ = 56.857, *p* < 0.001). The results of the simple slope test are shown in Figure 4. On the whole, solvers who pursued the availability goal scored higher than problem solvers who did not pursue the availability goal. This suggested that the availability goal was prioritized over the price goal. For those with high availability goal pursuit, price goal pursuit significantly positively predicted task score (*B* = 0.201, *p* < 0.001). However, for those with low availability goal pursuit, price goal pursuit significantly negatively predicted task score (*B* = −0.172, *p* < 0.001) (see Figure 4). These results indicated that, when solvers did not work hard to pursue the availability goal and pursued the price advantage blindly, their task score dropped significantly. If solvers worked hard toward achieving the availability goal pursued price advantage, they would achieve higher scores.

### Linear Regression of Goals Pursuit on CPS Proficiency

A linear regression model was built to examine goal pursuit as the predictor of CPS proficiency using the least-squares regression method. All of the model tests were significant, with *R*^2^ = 0.078. Demand goal pursuit (*B* = 20.700, *t* = 12.832, *p* < 0.001) and availability goal pursuit (*B* = 8.204, *t* = 5.234, *p* < 0.001) significantly positively predicted CPS proficiency (see Table 5).

**TABLE 5:** Linear regression coefficients for goal pursuit on CPS proficiency.

	**Model 1**				**Model 2**			
Predictor	*B*	*SE*	*t*	β	*B*	*SE*	*t*	β
Demand	20.585	1.491	13.808^∗∗∗^	0.237	20.700	1.613	12.832^∗∗∗^	0.239
Availability	9.450	1.558	6.064^∗∗∗^	0.109	8.204	1.568	5.234^∗∗∗^	0.095
Availability * Demand					4.345	1.647	2.637^∗∗^	0.050
Availability * price					7.329	1.380	5.309^∗∗∗^	0.096
*R*^2^	0.066^∗∗∗^				0.078^∗∗∗^			
Δ*R*^2^					0.012^∗∗∗^			

*^∗∗∗^*p* < 0.001. The variables not significant was excluded in the model. B stands regression coefficients, and β stands standardized regression coefficients.*

**FIGURE 5:**
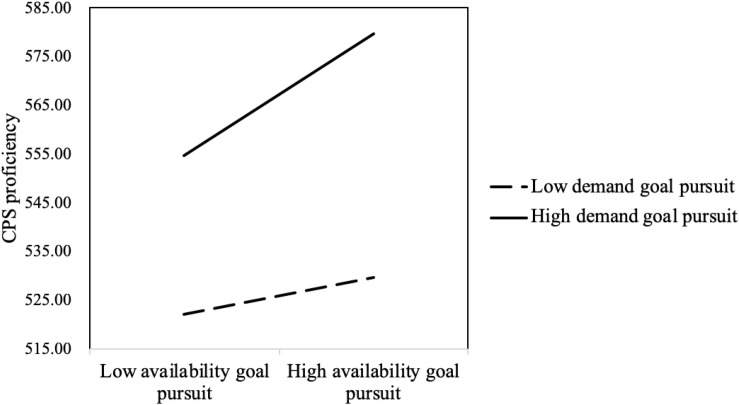
Interaction between availability goal pursuit and demand goal pursuit.

The interaction between demand goal pursuit and availability goal pursuit also significantly negatively predicted CPS proficiency (*B* = 4.345, *t* = 2.637, *p* < 0.01). The simple slope test showed that, for those with high demand goal pursuit, the availability goal significantly positively predicted CPS score (*B* = 12.557, *p* < 0.05), and for those with low demand goal pursuit, the availability goal did not significantly predict CPS score (*B* = 3.810, *p* = 0.058). Solvers who pursued the demand goal and the availability goal achieved the highest CPS proficiency scores (see Figure 5).

**FIGURE 6:**
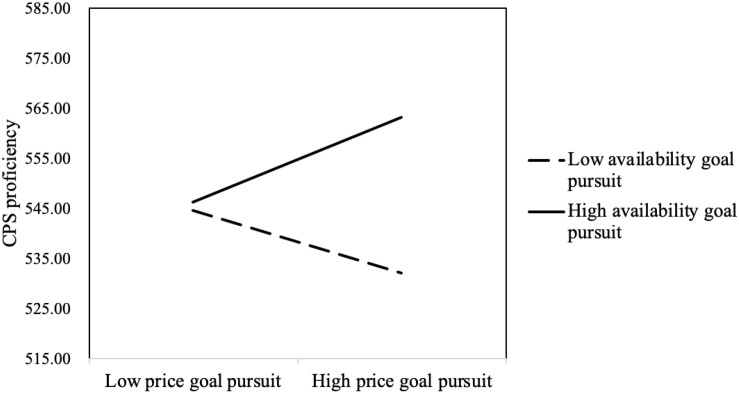
Interaction between availability goal pursuit and price goal pursuit.

The interaction between price goal pursuit and availability goal pursuit significantly positively predicted CPS proficiency (*B* = 7.329, *t* = 5.309, *p* < 0.001). The results of the simple slope test showed that, for those with high availability goal pursuit, price goal pursuit significantly positively predicted CPS proficiency (*B* = 8.411, *p* < 0.001); for those with low availability goal pursuit, price goal pursuit significantly negatively predicted CPS proficiency (*B* = −6.275, *p* < 0.01) (see Figure 6).

## Discussion

Although CPS assessment has been researched for more than 40 years, its development as an assessment tool is far from mature. Many researchers have proposed rich theoretical models of CPS, but none of these suggested models has been fully validated (Fischer et al., 2012; Greiff and Fischer, 2013; Greiff et al., 2013).

The present study explored multiple goals balancing behavior shown in the log data of the third subtask of the “Ticket” task of PISA 2012. The purpose of the study was to test and supplement the theoretical discussion of previous studies through an analysis of measured data. The main findings are described in two sections below.

### Coping Strategies in Multiple Goals Conflict Scenarios

#### The Importance of Clarifying Goals and Working Hard for Them

Dörner placed great emphasis on the importance of clarifying goals in a vague situation, noting that this is often the first step in solving a problem (Dörner and Wearing, 1995). The present study analyzed the log data of a CPS task to explore solvers’ ability to clarify and pursue each goal. In the task, solvers needed to first clarify the demand and price goals, then identify the availability goal following failure to secure a ticket. The demand and availability goals had to be met to solve the problem, but their difficulty levels obviously differed. The result showed that, on average, 90.12% of solvers’ plans met the demand goal, but only 61.91% met the availability goal. As solvers had to sacrifice the price goal to get the ticket, only 68.30% met this goal. In fact, unless goals were in direct conflict, solvers’ goal-oriented actions toward as many goals as possible resulted in more effective problem solving. For example, amongst solvers who gave up the demand goal and turned to pursue the availability goal, task scores were higher for those who also pursued the price goal than for those who ignored it.

#### The Importance of Prioritizing Goals and Balancing Strategies

In a CPS task with conflicting goals, solvers must do more than clarify goals to solve the problem; they must also correctly prioritize the goals and execute strategies to deal with conflicting goals. For this reason, Funke (2001) strongly emphasized the cognitive process of assessing and prioritizing goals in CPS.

In the task used in the present study, because tickets that met the ride demand and were also available were not concession tickets, solvers had to buy a slightly more expensive ticket in order to solve the problem. Therefore, the demand and availability goals were superior to the price goal, and solvers should have given priority to achieving the first two goals before striving to fulfill the price goal. The regression and cluster analyses showed that if solvers tried hard to pursue both goals, they solved the problem well; if they did not work hard toward one of the goals, they were almost unable to solve the problem. Fischer et al. (2012) suggested that, when there is a conflict between goals, solvers must find a satisfactory trade-off by only partially achieving some goals. This is indeed an effective strategy; it is very important to prioritize goals and pursue the most important ones, rather than all goals.

The results also showed that the demand goal resulted in a larger regression coefficient than that of the availability goal. Further, the cluster analysis demonstrated that solvers who pursued the demand goal scored highest and solvers who gave up the demand goal scored lowest. This suggested that, although the demand goal and the availability goal were both important, the demand goal had priority over the availability goal. Indeed, in the task, since “the availability of such a ticket” was unpredictable, solvers should have first ensured that each solution met the demand goal before working toward the availability goal. Effective solvers prioritized goals in the following sequence: demand goal, availability goal, and price goal. Thus, when solvers face multiple conflicting goals in a problem, they should first consider the most important goals and gradually explore ways to balance them and then consider the secondary goals. If the process is reversed, with the important goals sacrificed for the secondary goals, problem solving will fail.

### The Importance of Theoretically-Driven Log Data Analysis

In the present study, the extraction of goal pursuit indicators from the log data were based on the task feature of multiple goals conflict. The resulting indicators were meaningful and easy to facilitate the next step of analysis. This process of identifying indicators fundamentally differs from that of data-driven analysis, which commonly obtains indicators that are huge and uninterpretable. Researchers have to use complex data mining techniques such as machine learning or deep learning to analyze a large number of features or indicators in the data-driven analysis. Therefore, using an appropriate theory to analyze log data files is often a multiplier.

We used cluster and regression analysis to explore solvers’ goal prioritization and balancing strategies in the context of multiple goals conflict. The results strengthen our understanding of multiple goals balancing behavior and support and complement the theoretical elaboration of multiple goals balancing in CPS. They also demonstrate that theoretically-driven log data analysis cannot only make log analysis more concise, efficient, and interpretable, but also contribute to the confirmation, improvement, and promotion of CPS theory.

### Limitations

The present study analyzed the log data of a task with multiple conflicting goals of varying priorities. It demonstrated the importance and necessity of multiple goals balancing in CPS. However, there are still some shortcomings in this study, which should be supplemented and improved in further research.

Firstly, the findings of this study is only applicable to participants with three or more purchase attempts (3,201 students, 82.16% of all participants) because those with a lower number of purchase attempts were not included in the analyses. Secondly, this study used a subtask of the PISA test that was not designed originally for multiple goals balancing. Further research should develop more complex systems with multiple goals conflict in order to fully explore strategies of multiple goals balancing. These complex systems should involve an increasing number of complex goals, in order to better reveal how people solve problems effectively in complex problem scenarios. Otherwise, it should ensure that the complex system includes a more flexible “confirm submit” button. In such a scenario, every time a solver attempts a purchase plan, he/she will click a button to get information about the availability and price of the ticket. This will enable researchers to easily segment hidden plans, as the “confirm submit” button will automatically indicate the end of a complete plan in the log data.

## Conclusion

Overall, theoretically-driven log data analysis of CPS process data can extract valuable information from messy process data, and this information can contribute to the improvement of CPS cognitive theory. Competent solvers identify and clarify goals more effectively and ensure that each step of their action plan has a clear goal orientation. More importantly, successful coping with multiple goals in tasks requires proper goal prioritizing and balancing.

## Ethics Statement

The study did a secondary analysis of data, therefore: ethical review and approval was not required for the study on human participants in accordance with the local legislation and institutional requirements. Written informed consent from the participants was not required to participate in this study in accordance with the national legislation and institutional requirements.

## Author Contributions

FL designed the study, supervised the quality in the process of researching, and provided financial support. HL supervised the quality in the process of researching, revising it critically for important intellectual content, and provided financial support. XL collected and analyzed the data. YR analyzed the data. PR and DB wrote the manuscript.

## Conflict of Interest Statement

The authors declare that the research was conducted in the absence of any commercial or financial relationships that could be construed as a potential conflict of interest.

## Footnotes

1 The Program for International Student Assessment (PISA) is a comprehensive large-scale international comparative test project conducted by the Organization for Economic Cooperation and Development (OECD) (OECD Indicators, 2009).
